# CARE-RESISTANT BEHAVIOR TRAJECTORIES OF PERSONS WITH DEMENTIA IN NURSING HOMES AND DISTAL OUTCOMES IN ORAL HEALTH

**DOI:** 10.1093/geroni/igad104.0079

**Published:** 2023-12-21

**Authors:** Chunhong Xiao, Frank Puga, Carolyn Pickering, Cindy Cain, Maria Geisinger, Rita Jablonski

**Affiliations:** The University of Alabama at Birmingham, Northport, Alabama, United States; The University of Alabama at Birmingham, Northport, Alabama, United States; The University of Alabama at Birmingham, Northport, Alabama, United States; The University of Alabama at Birmingham, Northport, Alabama, United States; The University of Alabama at Birmingham, Northport, Alabama, United States; The University of Alabama at Birmingham, Northport, Alabama, United States

## Abstract

Persons with dementia (PWD) who exhibit care-resistant behaviors (CRB) are likely to resist mouthcare and have poor oral health. This study aimed to examine predictors and describe the characteristics of CRB trajectories and their distal outcome in oral health among PWD in nursing homes (NH). Group-based trajectory modeling was used to analyze dynamic changes in CRB intensity over 21 days with two time-points (morning and afternoon) among 75 PWD. Sub-group analysis demonstrated the characteristics of each trajectory group membership. Distal-outcome models explored the association between oral health variation and mouthcare completion to CRB trajectories. The most influential predictors included CRB intensity at baseline, duration of mouthcare, and the number of antipsychotics prescribed. Three distinctive CRB trajectories were identified for both morning and afternoon in the context of mouthcare. Oral health status and the mouthcare completion rates differed by CRB trajectory groups, and worse oral health status was associated with higher level of CRB intensity trajectories. The High-start CRB groups had the worst oral health status and lowest mouthcare completion in both model estimation and pre-post test. Oral health showed improvement across all three trajectory groups. A tailored individual-level strategy based on a daily pattern of CRB intensity may provide the key to developing interventions that find the “sweet spot” between providing optimal mouthcare while minimizing CRB. Results imply that balancing CRB and mouthcare by providing morning-only mouthcare to those with high CRBs may allocate resources to PLWD most in need of mouthcare. Antipsychotics have not shown usefulness in managing CRB.

